# A Molecular Low‐Coordinate [Fe‐S‐Fe] Unit in Three Oxidation States

**DOI:** 10.1002/chem.202100336

**Published:** 2021-03-05

**Authors:** Christian Schneider, Serhiy Demeshko, Franc Meyer, C. Gunnar Werncke

**Affiliations:** ^1^ Fachbereich Chemie Philipps-Universität Hans-Meerwein-Str. 4 35043 Marburg Germany; ^2^ Institut für Anorganische Chemie Universität Göttingen Tammannstr. 4 37077 Göttingen Germany

**Keywords:** ^57^Fe Mössbauer spectroscopy, electrochemistry, iron sulfide complex, magnetism, nitrogenase

## Abstract

A [Fe‐S‐Fe] subunit with a single sulfide bridging two low‐coordinate iron ions is the supposed active site of the iron‐molybdenum co‐factor (FeMoco) of nitrogenase. Here we report a dinuclear monosulfido bridged diiron(II) complex with a similar complex geometry that can be oxidized stepwise to diiron(II/III) and diiron(III/III) complexes while retaining the [Fe‐S‐Fe] core. The series of complexes has been characterized crystallographically, and electronic structures have been studied using, inter alia, ^57^Fe Mössbauer spectroscopy and SQUID magnetometry. Further, cleavage of the [Fe‐S‐Fe] unit by CS_2_ is presented.

## Graphical abstract

**A dinuclear monosulfido bridged diiron(II) complex** is reported that can be oxidized stepwise to diiron(II/III) and diiron(III/III) complexes while retaining the [Fe‐S‐Fe] core. This unprecedented series of low‐coordinate complexes has been thoroughly characterized using inter alia, ^57^Fe Mössbauer spectroscopy and SQUID magnetometry.

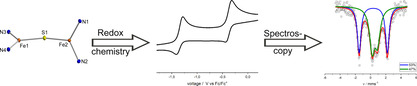

## Introduction

**Figure 1:**
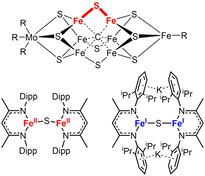
Top: FeMo co‐factor of the FeMo‐N_2_ase. Bottom: Sole examples of three‐coordinate [Fe‐S‐Fe] complexes. Dipp=2,6‐diisopropylphenyl.

Nitrogenase (N_2_ase) is an important enzyme that catalyses primarily the reduction of dinitrogen to ammonia. The reaction takes place at the iron‐molybdenum co‐factor (FeMoco), which constitutes a ligated [MoFe_7_S_9_C] unit.^[1]^ Despite tremendous advances in the structural elucidation of the co‐factor and some intermediate clusters under substrate conversion, as well as detailed studies of the co‐factor's electronic structures, the exact iron site for substrate binding and involved reaction mechanisms are still not fully understood.[^2^, ^3^] Increasing evidence points to the importance of the Fe‐S‐Fe belt unit (Figure 1, top).[^4^, ^5^]

This unit is assumed to open up during substrate turnover, as experimentally shown by displacement of the sulfide unit by CO or selenide.[^2^, ^6^] As such there is inherent interest in molecular models that feature an unsupported [Fe‐S‐Fe] unit with iron ions in a low‐coordinate environment. However, the only known examples bearing three‐coordinate metal ions are a β‐diketiminato (nacnac) based iron(II) complex as well as its two‐fold reduced iron(I) derivative (Figure 1, bottom).[^7^, ^8^] The iron(II) complex is susceptible to coordination by ammonia and hydrazines, and is even able to cleave the N−N bond of the latter.[^7^, ^9^] We now report on the synthesis of a dinuclear [2Fe‐1S]^2+^ complex with an unsupported monosulfide bridge via the reaction of a two‐coordinate iron(I) silylamide with elemental sulfur. Subsequent oxidation leads to the first example of a mixed valent [2Fe‐1S]^3+^ and an “all ferric” [2Fe‐1S]^4+^ form. The series of complexes was examined with respect to their spectroscopic and physical properties. The initial [2Fe‐1S]^2+^ complex was further subjected to a variety of small molecule substrates that are transformed by the N_2_‐ase, however showing only a limited reactivity or stability. Most notably, its reaction with CS_2_ led to rupture of the Fe‐S‐Fe motif and formation of a mononuclear iron(II) thiocarbonate complex, revealing the structural lability of the [Fe‐S‐Fe] unit.

## Results and Discussion

**Scheme 1:**
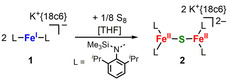
Synthesis of the monosulfide bridged complex **2**.

**Figure 2:**
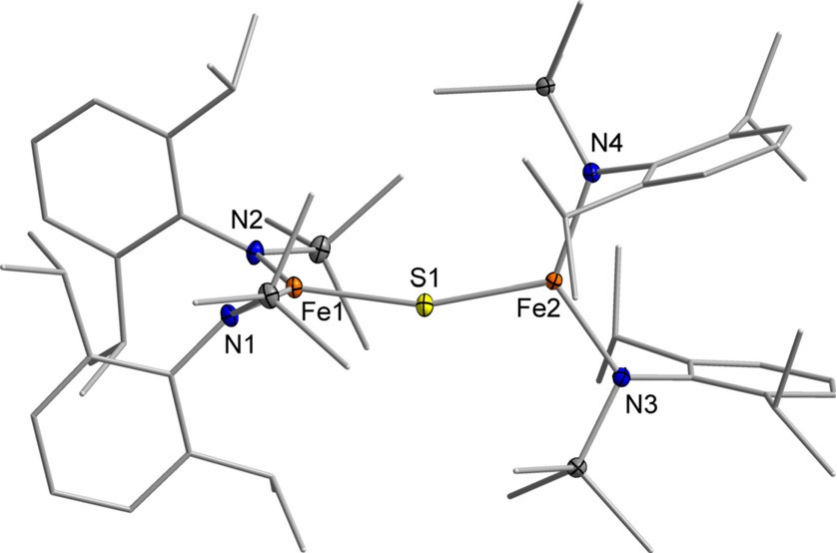
Molecular structure of **2** in the solid state. K^+^{18c6} counter ions and H atoms are omitted. See Table 1 for important bond lengths and angles.

The reaction of a suspension of K{18c6}[FeL_2_] (L=‐N(Dipp)SiMe_3_, Dipp=2,6‐diisopropylphenyl), **1**,^[10]^ in Et_2_O with 1/16 S_8_ for 16 h led to the formation of a colorless solid. X‐Ray diffraction analysis of suitable single crystals showed the formation of the dinuclear complex (K{18c6})_2_[(FeL_2_)_2_(μ‐S)], **2** (Scheme 1, Figure 2).

**2** features two three‐coordinate iron(II) ions bridged by a single sulfide in a nearly linear fashion (Fe‐S‐Fe 167.78(2)°) with slightly different Fe−S distances of 2.2400(5) Å and 2.337(5) Å (Table 1). The linear arrangement is unusual as in dinuclear metal complexes with an unsupported bridging S^2−^ ligand Fe−S−Fe bond angles of 90° to 140° are commonly found.[^7^, ^11^] A linear Fe‐S‐Fe axis was only observed in related [2Fe‐1S]^0, 2−^ complexes (Figure 1)[^7^, ^8^] and in a higher coordinate salen based compound,^[12]^ and was attributed to steric constraints. **2** represents only the second example of a dinuclear, low‐coordinate iron(II) complex bearing an unsupported sulfide bridge.^[7]^

**Table 1:** Selected bond lengths (Å) and angles (°) of complexes **2**–**4**.

Compound	**2**	**3**	**4**
Fe1−S1	2.2400(5)	2.1911(11)	2.1746(7)
Fe2−S1	2.337(5)	2.1943(11)	2.1739(7)
Fe1−N1	1.9961(13)	1.928(3)	1.8871(19)
Fe1−N2	1.9778(14)	1.927(3)	1.8902(19)
Fe2−N3	2.0123(13)	1.925(3)	1.886(2)
Fe2−N4	1.9871(13)	1.921(3)	1.8859(19)
Fe1‐S1‐Fe2	161.78(2)	175.2(7)	172.31(4)

**Figure 3:**
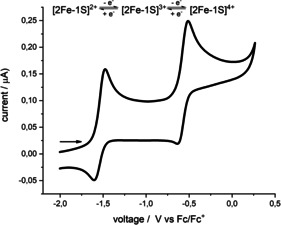
Cyclic voltammogram of **2** (200 mV s^−1^, THF, 0.1 m NBu_4_PF_6_, vs. Fc/Fc^+^.

As the iron ions in the FeMo co‐factor are supposed to switch between oxidation states +2 and +3 during substrate conversion^[13]^ we were interested if the oxidation state of **2** can be adjusted accordingly. The cyclic voltammogram of **2** in THF showed two quasi‐reversible one‐electron oxidation processes at *E*_1/2_=−1.55 V and −0.55 V (versus Fc/Fc^+^, Figure 3), which were tentatively assigned to the [2Fe‐1S]^2+^/^3+^ and the [2Fe‐1S]^3+/4+^ couple, respectively. Electrochemical data on the oxidation of a low‐coordinate [Fe^II/III^‐S‐Fe^II/III^] unit is absent in the literature.

The related bis(μ‐sulfido) diferric complex [(LFe)_2_(μ‐S)_2_] (monoanionic ligand L^−^=nacnac) reported by Driess and co‐workers features redox events in THF at *E*_1/2_=−1.45 V for the [2Fe‐2S]^2+^/^1+^ and *E*_1/2_=−2.55 V (Δ*E*_1/2_=1.10 V) for the [2Fe‐2S]^1+/0^ redox couple.^[14]^ For the similar dianionic complex [(LFe)_2_(μ‐S)_2_]^2−^ [dianionic ligand L^2−^=bis(benzimidazolato)), reported by some of us, the reduction of the diferric [2Fe‐2S]^2+^ core in MeCN occurred at *E*_1/2_=−1.14 V ([2Fe‐2S]^2+/1+^) and *E*_1/2_=−2.10 V ([2Fe‐2S]^1+/0^, Δ*E*_1/2_=0.96 V).^[15]^ The difference between their respective redox events is consistent with the one found for **2** (Δ*E*_1/2_=0.98 V). In contrast, the positions of the redox events of these bis(μ‐sulfido) complexes are shifted by around 0.9 V or 0.6 V to lower potentials, respectively, reflecting the additional sulfide ligation and higher coordination number of the iron ions in the two previously reported [2Fe‐2S] systems.[^14^, ^15^]

**Scheme 2:**
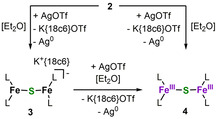
Synthesis of complexes **3** and **4** by stepwise oxidation of **2**.

**Figure 4:**
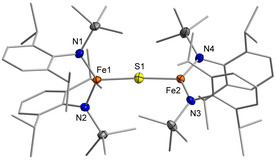
Molecular structure of **3** in the solid state. The K^+^{18c6} counter ion and H atoms are omitted. See Table 1 for important bond lengths and angles.

Given the electrochemical data we attempted the chemical oxidation of **2**. Treatment of **2** with one equivalent of AgOTf in Et_2_O yielded reddish K{18c6}[(L_2_Fe)_2_(μ‐S)], **3** (Scheme 2, Figure 4). Treatment of **2** with two equivalents of AgOTf in Et_2_O (or **3** with one equivalent of AgOTf) led to a colour change to dark green, and the neutral complex [(L_2_Fe)_2_(μ‐S)], **4**, was obtained from a saturated pentane solution.

Important structural parameters of **3** and **4** are shown in Table 1. Compared to **2**, complexes **3** and **4** retain both a more or less linear Fe‐S‐Fe core. A gradual decrease of the Fe−S and Fe−N bond lengths was observed upon oxidation due to the contraction of the ionic radii of the metal ions. The structural features found for both iron ions are largely identical in **3** and **4**, which prohibited an assignment of localized oxidation states for mixed‐valent **3** on a structural level.

**Figure 5:**
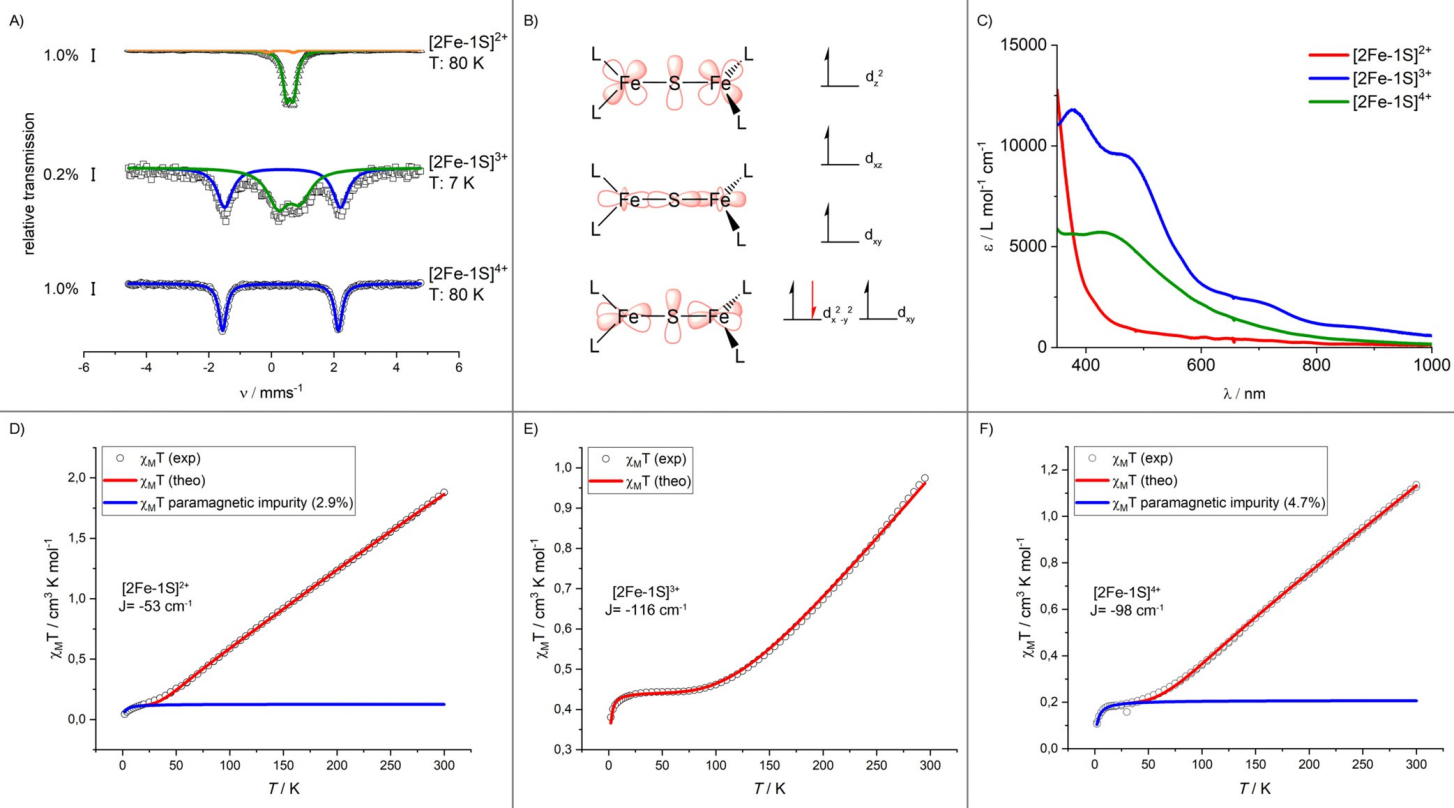
Spectroscopic and magnetic features of **2**–**4**. (A) Overlay of the UV/VIS spectra of **2** (red), **3** (blue) and **4** (green) in THF. (B) Zero‐field Mössbauer spectra of solid **2** (80 K), **3** (7 K) and **4** (80 K). Parameters for **2**: *δ*=0.59 mm s^−1^, |Δ*E*_Q_|=0.22 mm s^−1^; **3**: *δ_1_*=0.36 mm s^−1^, |Δ*E*_Q_|_1_=3.70 mm s^−1^ (blue), *δ_2_*=0.57 mm s^−1^, |Δ*E*_Q_|_2_=0.71 mm s^−1^ (green); **4**: *δ*=0.29 mm s^−1^, |Δ*E*_Q_|=3.70 mm s^−1^. (C) Simplified depiction of the (non)interacting d‐orbitals responsible for magnetic superexchange of a linear Fe‐S‐Fe arrangement (left), qualitative orbital splitting splitting diagram for each iron ion in a C_2V_ symmetric environment (right). The electron which is lost upon oxidation is shown in red. (D)‐(F) Variable‐temperature magnetic susceptibility of solid **2**–**4** in the range 2–300 K (B=0.5 T).

With this unprecedented series of low‐coordinate [2Fe‐1S] complexes in three different oxidation states in hand, their spectroscopic and electronic features were studied in detail. UV/Vis spectroscopic examination of **2** showed no absorption beyond 400 nm (Figure 5 A), which is common for low‐coordinate iron(II) compounds,^[16]^ but unusual for μ‐sulfido diiron(II) complexes.[^7^, ^15^] In contrast, the mixed valent species **3** exhibited three rather intense maxima at 380 nm (*ϵ*=11 800 L mol^−1^ cm), 470 nm (*ϵ*=9540 L mol^−1^ cm) and 700 nm (*ϵ*=2270 L mol^−1^ cm), and it is tempting to assign the low‐energy absorption to an intervalence charge transfer (IVCT) transition. For **4** just one pronounced band at 430 nm (*ϵ*=5710 L mol^−1^ cm) was observed. Zero field ^57^Fe Mössbauer spectroscopy (Figure 5 B) revealed for the iron(II/II) complex **2** a doublet with *δ*=0.59 mm s^−1^ and |Δ*E*_Q_|=0.22 mm s^−1^, in agreement with data for a related three‐coordinate iron(II) complex.^[17]^ These values are slightly smaller than those of the only other known monosulfido‐bridged iron(II/II) complex shown in Figure 1 (bottom left; *δ*=0.86 mm s^−1^ and |Δ*E*_Q_|=0.58 mm s^−1^), which can be explained by the weaker donor strength of the silylamide ligands as well as a less distorted trigonal planar ligand arrangement.^[7]^ The all ferric complex **4** is represented by a doublet with *δ*=0.29 mm s^−1^ and |Δ*E*_Q_|=3.70 mm s^−1^ indicating the presence of high‐spin iron(III) ions. The spectrum of the mixed valent complex **3**, recorded at 7 K, showed two doublets with *δ*=0.36 mm s^−1^ (|Δ*E*_Q_|=3.70 mm s^−1^) and *δ*=0.57 mm s^−1^ (|Δ*E*_Q_|=0.71 mm s^−1^). This evidences distinguishable iron(II/III) positions in solid **3** on the ^57^Fe Mössbauer timescale at 7 K, whereas the smaller separation in the isomer shifts (Δ*δ*(**3**)=0.21 mm s^−1^ vs. Δ*δ*(**2**/**4**)=0.30 mm s^−1^) is indicative of some degree of valence delocalisation. Given the lack of literature precedence of the three‐coordinate μ‐sulfido complexes **3** and **4** their Mössbauer spectroscopic features are compared best to low coordinate iron complexes bearing a [2Fe‐2S] motif in the same oxidation states.[^14^, ^15^, ^18^, ^19^, ^20^] Most importantly, such mixed valent [2Fe‐2S] compounds are shown to exhibit moderate[^15^, ^19^, ^20^] or strong^[14]^ antiferromagnetic coupling, and give Mössbauer spectra (at <10 K) that correspond to either valence localized or delocalized states, respectively.

Additional insights into the electronic situation of **2**–**4** in solid state were obtained by SQUID measurements (Figure 5 D–F). **2** exhibited at 300 K a *χ*T value of 1.8 cm^3^ mol^−1^ K which linearly dropped to ca. 0.1 cm^3^ mol^−1^ K at 30 K. This indicated a moderate antiferromagnetic interaction between the two iron(II) (*S*=2) ions with a *S*=0 ground state. The coupling constant was determined to be *J*=−53 cm^−1^ using *Ĥ*=−2*J*S_A_⋅S_B_ with g_1_=g_2_=2.01.

The mixed valent compound **3** showed at 295 K a significantly lower *χ*T value of 0.96 cm^3^ mol^−1^ K which decreased to 0.44 cm^3^ mol^−1^ K at 80 K with a further drop to 0.38 cm^3^ mol^−1^ K below 20 K, which implies a ground state of *S*=1/2. The antiferromagnetic coupling is stronger with *J*=−115 cm^−1^ (g_1_=2.08, g_2_=2.02). For the all‐ferric compound **4** a similar value *J*=−104 cm^−1^ (g_1_=g_2_=2.10, S_1_=S_2_=5/2) was observed with *χ*T=1.3 cm^3^⋅mol^−1^⋅K at 300 K that decreased linearly to ca. 0.2 cm^3^ mol^−1^ K below 50 K due to a *S*=0 ground state. The differences in exchange coupling can be explained using a simplified orbital scheme under assumption of an idealized C_2V_ symmetric ligand environment for each iron atom (Figure 5 C, *z*‐axis along the Fe‐S‐Fe unit). Upon oxidation, electrons are removed from the lowest‐lying, co‐parallel *d*_xy_/*d*_x2‐y2_ orbitals, which have no impact onto the exchange mechanism. As such the variation in *J* values for **2**–**4** can be mainly attributed to differences in Fe⋅⋅⋅Fe distances, with different superexchange contributions due to changes in Fe‐(*μ*‐S) covalency likely playing a further role. A significant stabilization of the antiferromagnetically coupled ground state upon oxidation from the diiron(II) to the mixed‐valent iron(II)/iron(III) and diiron(III) states was observed for the series of complexes [(LFe)_2_(μ‐S)_2_]^4−/3−/2−^ (L^2−^=bis(benzimidazolato)).[^15^, ^19^]

**Scheme 3:**
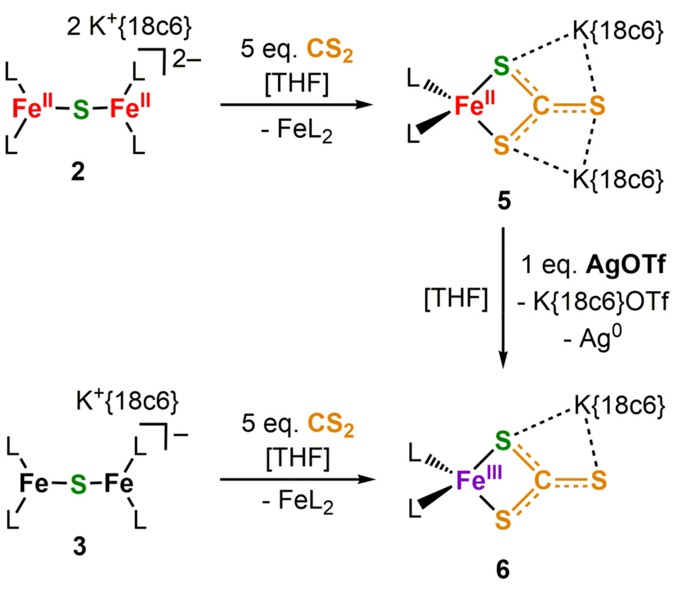
Reaction of **2** and **3** with CS_2_ giving the thiocarbonate complexes **5** and **6**.

**Figure 6:**
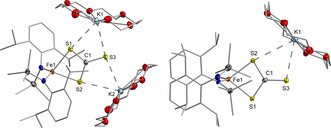
Molecular structures of **5** (left) and **6** (right) in the solid state. H atoms and THF molecules coordinating to each potassium ion are omitted. Selected distances (Å) and angles (°): **5**: Fe−S1 2.4504(5), Fe−S2 2.4275(5), Fe−N1 2.014(1), Fe−N2 2.012(1), N1‐Fe1‐N2 129.81(6), S1‐Fe1‐S2 72.86(1); **6**: Fe‐S1 2.3717(8), Fe‐S2 2.3835(9), Fe‐N1 1.917(2), Fe‐N2 1.917(2), N1‐Fe1‐N2 130.50(1), S1‐Fe1‐S2 75.88(3).

Having evaluated the redox and electronic properties of **2** we continued with investigations concerning its reactivity towards nitrogenase related small molecules.^[21]^ No reaction with N_2_, H_2_, or CO was observed whereas treatment of **2** with hydrazine derivatives and proton or methyl group sources only led to decomposition. Exposure of **2** to CO_2_ caused a visible colour change but did not yield any identifiable product, probably due to parallel insertion of CO_2_ into the iron silylamide bonds.^[22]^ As such we examined the behavior of **2** towards the heavier congener CS_2_, which is an inhibitor of nitrogenase‐mediated proton or acetylene reduction but can also serve as a substrate that is mainly converted to H_2_S.[^23^, ^24^, ^25^] This led to the isolation of the monomeric iron thiocarbonate complex (K{18c6})_2_[Fe(η^2^‐CS_3_)L_2_], **5** (Scheme 3, Figure 6).

In **5** the iron ion is coordinated by two silylamides and one bidentate thiocarbonate ligand in a distorted tetrahedral fashion. The two K^+^{18c6} cations are connected further to the thiocarbonate ligand, each via two sulfur atoms. We explain the formation of **5** by initial insertion of CS_2_ into one of the iron‐sulfur bonds of **2**, giving a thiocarbonate bridged dimer. The insertion of CS_2_ into an unsupported M‐S‐M unit was so far only reported for diuranium complexes.^[26]^ One neutral iron(II) bisamide is then replaced by the potassium crown‐ether moieties, which themselves act as a Lewis acid. The mixed valent complex **3** reacted with CS_2_ also under rupture of the [Fe‐S‐Fe] motif. This yielded the iron(III) thiocarbonate complex **7**, which could alternatively be obtained via the oxidation of **6** by silver triflate. For the neutral complex **4** the reaction with CS_2_ remained inconclusive. The observation of facile Fe–S bond cleavages suggests a rather weak Fe^II^–S interaction. The displacement of an iron(II) ion by other Lewis acids has possible implications for the situation found in the FeMo cofactor where cleavage of the belt Fe‐S‐Fe unit is discussed during substrate turnover using the local Lewis acid/base properties of the surroundings of the enzyme pocket.^[5]^ The facile insertion of CS_2_ into a [Fe‐S‐Fe] function also reveals how CS_2_ might act as an inhibitor of nitrogenase FeMoco (and other iron‐sulfur clusters) which is thought to proceed by blocking of coordination sites[^24^, ^25^, ^27^] or by insertion into other metal–ligand bonds.^[28]^

## Conclusions

We have synthesized a unique series of low‐coordinate [Fe‐S‐Fe] complexes in three oxidation states which resembles a Fe‐S‐Fe belt unit in the iron/sulfur/molybdenum co‐factor of the nitrogenase enzyme. These complexes were characterized for their magnetic and spectroscopic properties. ^57^Fe Mössbauer spectroscopy showed for the mixed valent [2Fe‐1S]^3+^ complex localized valence states in solid state at low temperatures. Magnetic measurements revealed for the diferrous [2Fe‐1S]^2+^ a moderate antiferromagnetic coupling which becomes significantly enhanced for the [2Fe‐1S]^3+^ and [2Fe‐1S]^4+^ compounds. Reactivity studies on these complexes towards different nitrogenase relevant substrates revealed for CS_2_ the facile cleavage of the Fe‐S‐Fe unit. This led to the formation of an iron thiocarbonate which may suggest a possible inhibitory mechanism of CS_2_ with respect to the reactivity of FeMoco and related Fe/S clusters.

## Experimental Section

General considerations: All manipulations were carried out in a glovebox, or using Schlenk‐type techniques under a dry argon atmosphere. Used solvents were dried by continuous distillation over sodium metal for several days, degassed via three freeze‐pump cycles and stored over molecular sieves 4 Å. K{18c6}[FeL_2_] was synthesized according to the literature procedure. For details concernining data acquisition of solution and solid‐state analyses (^1^H‐NMR spectra, X‐ray diffraction analysis, cyclovoltametry, magnetic measurements and Mössbauer spectra), see the Supporting Information.

### Syntheses

**[K{18 c6}]_2_[(FeL_2_)_2_(μ‐S)] (2)**: [K{18c6}][FeL_2_], **1**, (267 mg, 0.31 mmol, 2 equiv) was suspended in 5 mL of Et_2_O. The slow addition of elemental sulfur (5.0 mg, 0.16 mmol, 1 equiv) led to an immediate colour change of the solution from red to brown and the precipitation of a pale yellow solid. Decanting off the supernatant, washing the residue with 2×5 mL of pentane and drying under reduced pressure afforded the crude product as a pale yellow crystalline solid. Recrystallization in THF/ pentane at −35 °C led to colourless crystals of **2** (141 mg, 0.08 mmol, 52 %), suitable for X‐ray diffraction. ^1^H‐NMR (500.1 MHz, [D_8_]THF, 300 K): *δ*=14.23, 9.30, 3.50, 1.96, −0.94, −1.70 ppm. Evans: (500.1 MHz, [D_8_]THF + 1 % SiMe_4_, 300 K): *μ_eff_*=3.98 *μ*_B_.FT‐IR (ATR): (cm^−1^): *v̄*=2896 (w), 1418 (w), 1352 (w), 1314 (w), 1235 (m), 1192 (w), 1103 (vs.), 962 (w), 907 (s), 835 (vs.), 775 (s), 664 (w), 529 (w), 423 (m). CHNS: calc. (C_84_H_152_Fe_2_K_2_N_4_O_12_Si_4_S 1744.44 g mol^−1^): C 57.84 H 8.78 N 3.21 S 1.84 found: C 57.93 H 8.69 N 3.58 S 1.36.

**[K{18 c6}][(FeL_2_)_2_(μ‐S)] (3)**: [K{18c6}]_2_[(FeL_2_)_2_(μ‐S)], **2**, (150 mg, 0.086 mmol, 1 equiv) was suspended in 5 mL of Et_2_O. Upon the addition of AgOTf (22 mg, 0.086 mmol, 1 equiv) the pale yellow suspension turned into a red solution with beginning precipitation of a dark solid (elemental silver). After stirring for 2 hours, the mixture was filtered, the residue washed with 2×3 mL Et_2_O and the combined filtrates were layered with 5 mL of pentane. Storing the solution at −35 °C for several days yielded to a dark red crystalline solid, suitable for X‐ray diffraction analysis. Decanting off the supernatant, washing the residue with 2×5 mL of pentane and drying under reduced pressure afforded [K{18c6}][(FeL_2_)_2_(μ‐S)], **3**, as a dark red crystalline solid (40 mg, 0.023 mmol, 33 %). The aforementioned procedure to synthesize 3 leads to a pure product according to elemental analysis (vide infra). To obtain a magnetically pure sample several recrystallization steps in Et_2_O/pentane were required, which led to a decrease of the yield to less than 10 %. ^1^H‐NMR: (500.1 MHz, [D_8_]THF, 300 K): *δ*=14.58, 14.10, 3.26, 3.07, −0.92, −2.01 ppm. Evans: (300.3 MHz, [D_8_]THF + 1 % SiMe_4_, 300 K): *μ_eff_*=3.70 *μ*_B_. FT‐IR (ATR): (cm^−1^): *v̄*=2954 (w), 1456 (w), 1421 (m), 1353 (w), 1309 (w), 1232 (s), 1179 (s), 1101 (vs.), 961 (m), 896 (m), 832 (vs.), 781 (vs.), 733 (s), 673 (s), 637 (w), 541 (m), 434 (s). UV/VIS (THF): λ/ nm (*ϵ*/ L mol^−1^ cm)=380 (11 800), 470 (9540), 700 (2270). CHNS: calc. (C_72_H_128_Fe_2_K_1_N_4_O_6_Si_4_S 1441.03 g mol^−1^): C 60.01 H 8.95 N 3.89 S 2.22 found: C 59.66 H 8.64 N 4.07 S 1.51.

**[(FeL_2_)_2_(μ‐S)] (4)**: [K{18c6}]2[(FeL_2_)_2_(μ‐S)], **2**, (150 mg, 0.086 mmol, 1 equiv) was suspended in 5 mL of Et_2_O. Upon the addition of AgOTf (44 mg, 0.172 mmol, 2 equiv) the pale yellow suspension turned into a dark green solution and a dark precipitate. After stirring for 2 hours, the mixture was filtered, the residue washed 2 times with 3 mL of Et_2_O and the combined filtrates were dried in vacuo. The residue was extracted with 5 mL of pentane. The solution was concentrated to 0.5 mL in vacuo and stored at −30 °C for several days. This resulted in the deposition of a dark green crystalline solid, suitable for X‐ray diffraction. Decanting off the supernatant and drying under reduced pressure afforded [(FeL_2_)_2_(μ‐S)], **4**, as a dark green crystalline solid (52 mg, 0.046 mmol, 53 %). ^1^H‐NMR: (500.13 MHz, [D_8_]THF, 300 K): *δ*=64.37, 34.82, 23.62, −0.89, −26.88 ppm. Evans: (300.3 MHz, [D_8_]THF + 1 % SiMe_4_, 300 K): *μ_eff_*=7.26 *μ*_B_. FT‐IR (ATR): (cm^−1^): *v̄*=2958 (m), 1423 (w), 1360 (w), 1310 (w), 1242 (s), 1169 (m), 1100 (s), 1024 (s), 908 (m), 826 (vs.), 783 (vs.), 728 (vs.), 678 (s), 632 (m), 535 (s), 436 (s). UV/VIS (THF): λ/ nm (*ϵ*/ L mol^−1^ cm)=430 (5710). CHNS: calc. (C_60_H_104_Fe_2_N_4_Si_4_S 1137.61 g mol^−1^): C 63.35 H 9.22 N 4.94 S 2.83 found: C 63.97 H 8.75 N 5.42 S 2.65.

**[K(18‐crown‐6]_2_[L_2_Fe^II^(η^2^‐CS_3_)] (5)**: [K{18c6}]_2_[(FeL_2_)_2_(μ‐S)], **2**, (50 mg, 0.029 mmol, 1 equiv) was dissolved in 2 mL of THF. The slow addition of CS_2_ (8.8 μl, 0.145 mmol, 5 equiv) led to a colour change of the solution from brown to clear orange. After stirring for 2 hours, the mixture was filtered and the filtrate layered by 20 mL of pentane. Storing the solution at −35 °C yielded to the precipitation of orange crystals, suitable for X‐ray diffraction. Decanting off the supernatant, washing of the residue with 2×5 mL of pentane and drying in vacuo afforded **5** as a dark orange crystalline solid (23 mg, 0.013 mmol, 55 %). ^1^H‐NMR: (500.1 MHz, [D_8_]THF, 300 K): no identifiable signals besides for K{18c6}. FT‐IR (ATR): (cm^−1^): *v̄*=2954 (m), 2892 (m), 1453 (m), 1422 (m), 1350 (m), 1311 (w), 1234 (s), 1188 (m), 1104 (vs.), 1054 (m), 961 (s), 920 (s), 881 (m), 834 (vs.), 777 (s), 744 (m), 664 (m), 533 (m), 434 (m), 407 (w). CHN: calc. (C_55_H_101_Fe_2_K_2_N_2_O_12_Si_2_S_3_⋅THF 1268.81 g mol^−1^): C 52.89 H 8.19 N 2.09 S 7.18 found: C 52.39 H 7.91 N 2.60 S 6.73.

**[K(18 c6]_2_[L_2_Fe^III^(η^2^‐CS_3_)] (6)**: [K(18c6]_2_[L_2_Fe^II^(η^2^‐CS_3_)], **5**, (87 mg, 0.69 mmol, 1 equiv) was dissolved in 2 mL of THF. Upon the addition of AgOTf (17.6 mg, 069 mmol, 1 equiv) the solution turned dark red and the precipitation of a grey solid was observable. After stirring for 2 hours, the mixture was filtered and the filtrate was layered with 2 mL of pentane. Storing the solution at −35 °C for several days led to the precipitation of dark red crystals, suitable for X‐ray diffraction. Decanting off the supernatant, washing of the residue with 2×5 mL of pentane and drying in vacuo afforded **6** as a dark red crystalline solid. K(18c6)OTf is the major side product of the reaction. As it has almost the same solubility as **6** in Et_2_O and THF, it was impossible to obtain an analytically pure sample of **6** upon recrystallization. Therefore **6** could only be characterized by X‐ray diffraction. It exhibits no identifiable ^1^H NMR spectroscopic signature.

Deposition Numbers 2048098 (**2**), 2048164 (**3**), 2048165 (**4**), 2048166 (**5**) and 2048167 (**6**) contain the supplementary crystallographic data for this paper. These data are provided free of charge by the joint Cambridge Crystallographic Data Centre and Fachinformationszentrum Karlsruhe Access Structures service www.ccdc.cam.ac.uk/structures.

## Conflict of interest

The authors declare no conflict of interest.

## Supporting information

As a service to our authors and readers, this journal provides supporting information supplied by the authors. Such materials are peer reviewed and may be re‐organized for online delivery, but are not copy‐edited or typeset. Technical support issues arising from supporting information (other than missing files) should be addressed to the authors.

Supplementary
