# Supplementary material for: A Molecular Low‐Coordinate [Fe‐S‐Fe] Unit in Three Oxidation States
Source: Chemistry. 2021 Mar 5;27(20):6348–53. doi: 10.1002/chem.202100336 (PMC8048577; doi:10.1002/chem.202100336)
Supplement: Supplementary file 1 — Supplementary [file CHEM-27-6348-s001.pdf]

Chemistry–A European Journal

Supporting Information

A Molecular Low-Coordinate [Fe-S-Fe] Unit in Three Oxidation States

Christian Schneider,[a] Serhiy Demeshko,[b] Franc Meyer,[b] and C. Gunnar Werncke*[a]

Table of Contents

General considerations 3

1. Synthesis $[K\{18c6\}]_2[(FeL_2)_2(\mu\text{-}S)]$ (2) .... **Fehler! Textmarke nicht definiert.**
2. Synthesis of $[K\{18c6\}][(FeL_2)_2(\mu\text{-}S)]$ (3) .... **Fehler! Textmarke nicht definiert.**
3. Synthesis of $[(FeL_2)_2(\mu^2\text{-}S)]$ (4) .... **Fehler! Textmarke nicht definiert.**
4. Synthesis of $[K(18\text{-crown-}6]_2[L_2Fe^{II}(\eta^2\text{-}CS_3)]$ (5) .... **Fehler! Textmarke nicht definiert.**
5. Synthesis of $[K(18c6]_2[L_2Fe^{III}(\eta^2\text{-}CS_3)]$ (6) .... **Fehler! Textmarke nicht definiert.**
6. Cyclic voltammetry 5
7. Mössbauer spectroscopy 8
8. Magnetic Susceptibility Measurement 10
9. X-Ray diffraction analysis and molecular structures 12
10. References 18

General considerations

All manipulations were carried out in a glovebox, or using Schlenk-type techniques under a dry argon atmosphere. Used solvents were dried by continuous distillation over sodium metal for several days, degassed via three freeze-pump cycles and stored over molecular sieves 4 Å. The ^{1}H NMR spectra were recorded on a *Bruker AV 500* NMR spectrometers. Chemical shifts are reported in ppm relative to the residual proton signals of the solvent (for ^{1}H) or relative to the signal of the solvent itself (^{13}C). IR measurements were conducted on a *Bruker Alpha ATR-IR* spectrometer. Elemental analysis was performed by the "in-house" service of the Chemistry Department of the Philipps University Marburg, Germany using a CHN(S) analyzer vario MICRO Cube (*Elementar)*.

Solution magnetic susceptibilities were determined by the Evans method[1] Anhydrous $FeCl_2$, $HN(SiMe_3)(Dipp)$, n-butyl lithium (2.5 M in hexane), 18-crown-6, tetramethylsilane (TMS), S_8, CS_2 were obtained commercially (Sigma-Aldrich, Acros, Strem, Alfa Aesar) and - if not noted otherwise - used as received. CS_2 was degassed, transferred into the glovebox and stored over molecular sieves. 18-crown-6 (18c6) was sublimed prior use to remove traces of water. LiL (L = $N(SiMe_3)Dipp$)[2], FeL_2[3] K{18c6}[FeL_2][4] were prepared according to literature procedures.

^{1}H-NMR-spectra

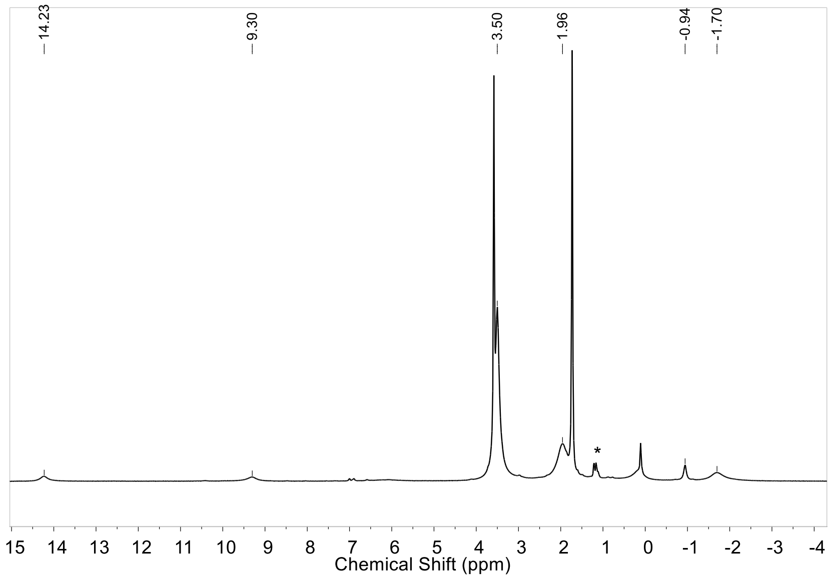

Figure S1. ^{1}H-NMR spectrum of $[2Fe–1S]^{2+}$ (**2**) in THF-d_8 (500.1 MHz). (*) denotes minor impurities of Et_2O and free ligand.

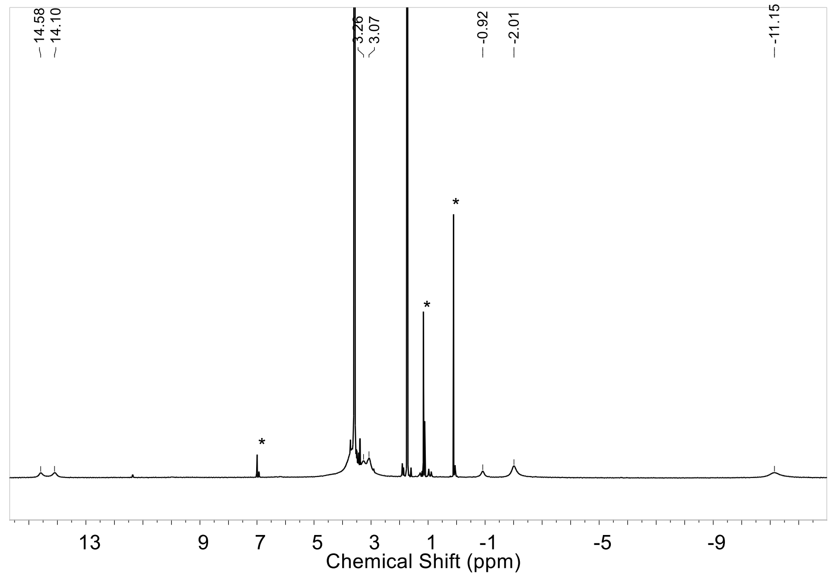

Figure S2. ^{1}H-NMR spectrum of $[2Fe–1S]^{3+}$ (**3**), in THF-d_8 (500.1 MHz). (*) denotes minor impurities of Et_2O and free ligand.

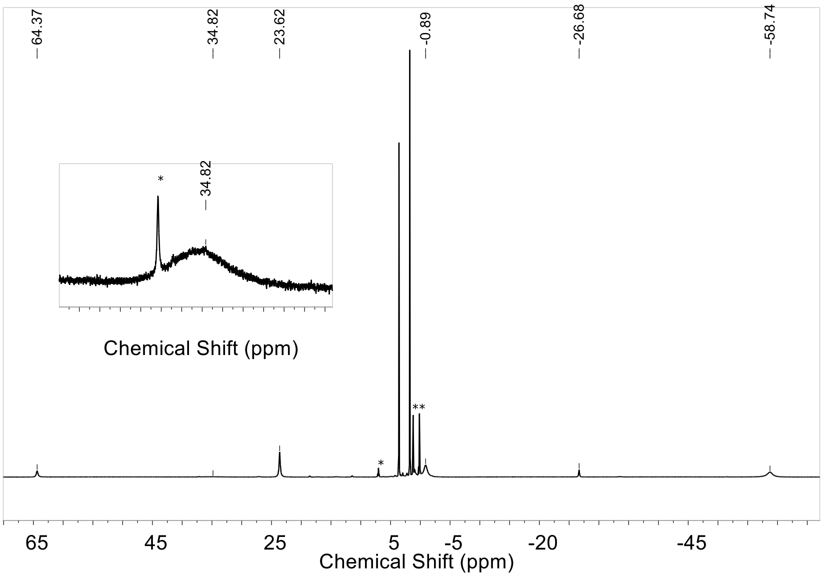

Figure S3. ^{1}H-NMR spectrum of $[2Fe–1S]^{4+}$ (**4**) in THF-d_8 (500.1 MHz). (*) denotes minor amounts of Et_2O, free ligand and an unknown paramagnetic impurity.

Cyclic voltammetry

The redox behavior of **2** was examined by cyclic voltammetry. A microcell HC "closed" stand (rhd instruments) was used in combination with a temperature controller (rhd instruments) and an AUTOLAB PGSTAT 204 (Metrohm GmbH) potentiostat/galvanostat. The measurements were performed at 25±0.1 °C, using a TSC 1600 Closed (rhd instruments) Pt cell in a three-electrode configuration with Pt wires acting as pseudo reference and as working electrode. To secure reproducible conditions the electrodes were freshly polished, rinsed with THF and dried *in vacuo* for 2 hours. 2 mM of analyte and 0.1 M $^{n}Bu_4N[PF_6]$, which acted as electrolyte, were used in the default measurement setup. The $[FeCp_2]$ / $[FeCp_2]^+$ (Fc/Fc^+) redox couple was utilized as internal standard. The measurements were performed at four different scan rates (50, 100, 200, 300, 400 and 500 mV/s), with two full cycles per scan rate. However, due to the fast decomposition of **2** under the measurement conditions it was not possible to get reliable voltammograms at scan rates < 200 mV/s. Peak potentials and currents of the second cycle of each measurement were determined using the NOVA Software (ver. 1.10.1.9, Metrohm GmbH).

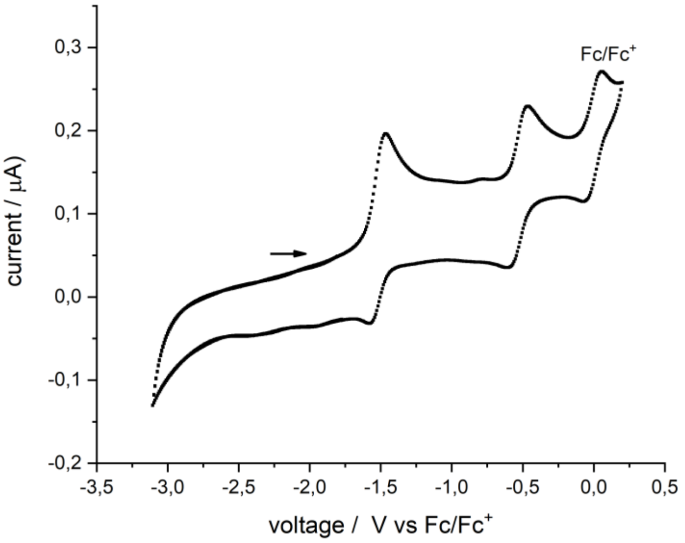

Figure S4. Cyclic voltammogram of $[2Fe-1S]^{2+}$ (2) in THF at a scan rate of 200 mV/s (0.1 M NBu_4PF_6, vs. Fc/Fc^+) in the rpesence of ferrocene.

The cyclic voltammogram **2** in THF shows two redox processes at $E_{1/2} = -1.55$ V and −0.55 V (versus Fc/Fc^+, which can assigned to the $[2Fe-1S]^{2+}/^{3+}$ and the $[2Fe-1S]^{3+/4+}$ couple (Figure S4). To characterize the electron transfers further Randles−Sevcik fit was used to verify if the analyt is freely diffusing or adsorbed at the electrode surface. The linear dependence of peak current on the square-root of the scan rate (Figure S5, Figure S6) points towards a diffusion controlled process for the electron transfers at $E_{1/2} = -1.55$ V and $E_{1/2} = -0.55$ V, respectively.

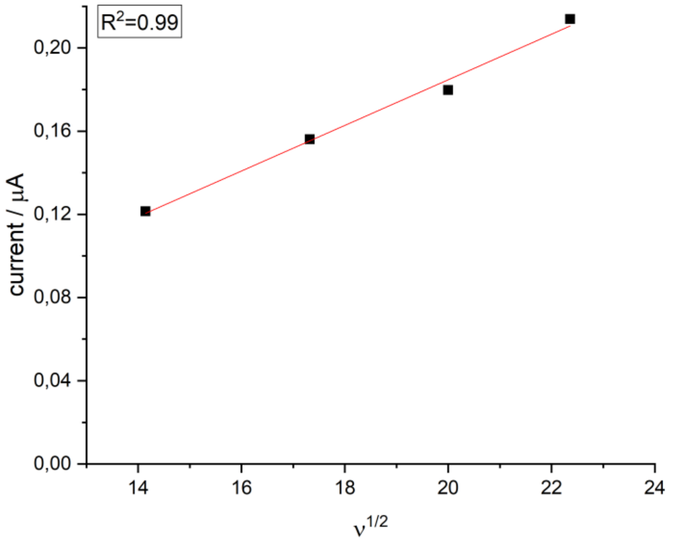

Figure S5. Randles−Sevcik fit for the electron transfer at $E_{1/2} = -1.55$ V.

Furthermore the ratio between the anodic and the cathodic peak current is greater than 1 for both electron transfers (**Table S1**, **Table S2**), indicating processes which may be coupled to a subsequent

chemical reaction. The peak separation is for both redox events greater than 50 mV implying quasi-reversible processes.

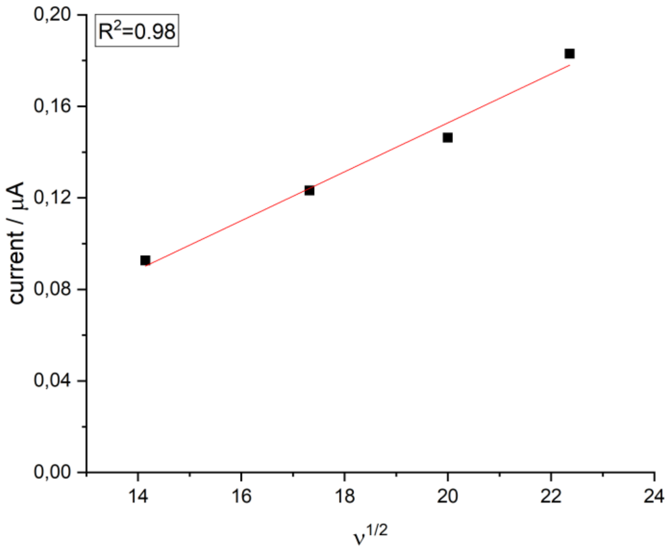

Figure S6: Randles–Sevcik fit for the electron transfer at $E_{1/2}$ = −0.55 V. The linear fit (red line) was calculated using the function $f(x) = mx + n$ and the least squares approach.

Table S1. Electrochemical data for the electron transfer at E1/2 = −1.55 V.

scan rate / mV/s	$E_{1/2}$ / V	I_{pa} / µA	I_{pc} / µA	i_{pa}/i_{pc}
200	-1.55	0.0926	-0.0665	1.39
300	-1.54	0.1318	-0.0862	1.53
400	-1.53	0.1462	-0.1043	1.40
500	-1.52	0.1830	-0.1243	1.47

Table S2. Electrochemical data for the electron transfer at $E_{1/2}$ = −0.55 V.

scan rate / mV/s	$E_{1/2}$ / V	I_{pa} / µA	I_{pc} / µA	i_{pa}/i_{pc}
200	-0.55	0.1214	-0.0752	1.61
300	-0.55	0.1560	-0.0862	1.81
400	-0.55	0.1797	-0.1516	1.18
500	-0.53	0.2138	-0.1771	1.21

Mössbauer spectroscopy

Mössbauer spectra were recorded with a ^{57}Co source in a Rh matrix using an alternating constant acceleration *Wissel* Mössbauer spectrometer operated in the transmission mode and equipped with a *Janis* closed-cycle helium cryostat. Isomer shifts are given relative to iron metal at ambient temperature. Simulation of the experimental data was performed with the *Mfit* program using *Lorentzian* line doublets: E. Bill, Max-Planck Institute for Chemical Energy Conversion, Mülheim/Ruhr, Germany.

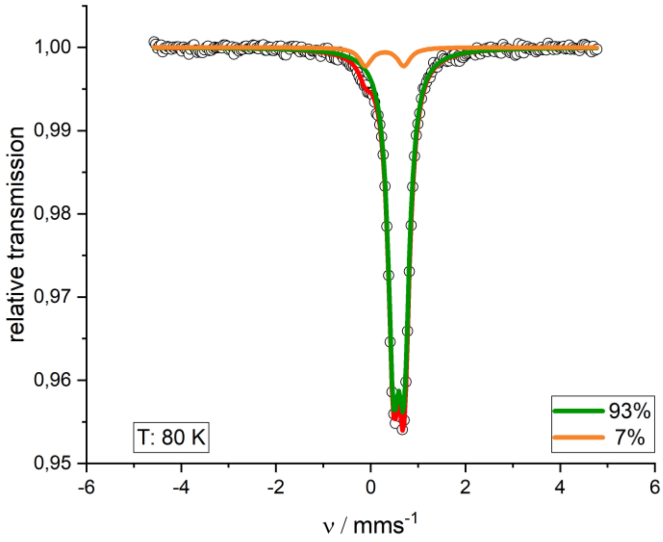

Figure S7. Zero field ^{57}Fe Mössbauer spectrum of solid $[2Fe–1S]^{2+}$ (**2**) at 80 K. The green line represents a fit with δ = 0.59 mm/s, $|\Delta E_Q|$ = 0.22 mm/s, which can be assigned to **2**. The orange line represents a fit with δ = 0.29 mm/s, $|\Delta E_Q|$ = 0.81 mm/s, which can be assigned to an unknown decomposition product.

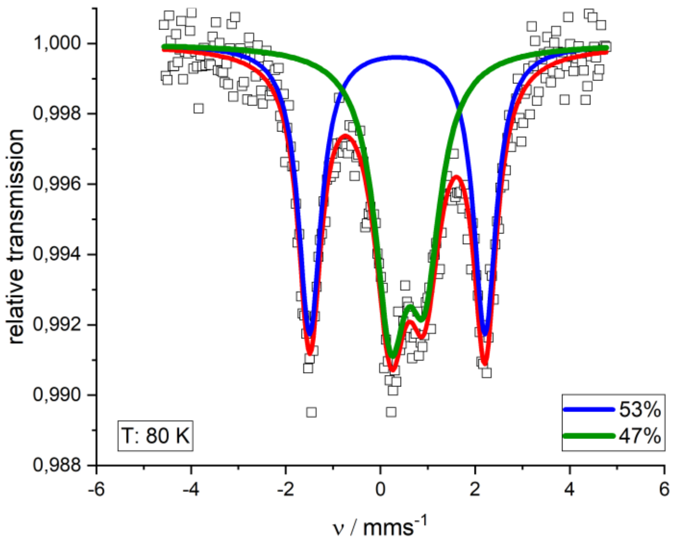

Figure S8. Zero field ^{57}Fe Mössbauer spectrum of solid $[2Fe–1S]^{3+}$ (**3**) at 7 K. δ_1 = 0.36 mm/s, $|\Delta E_Q|_1$ = 3.70 mm/s (blue), δ_2 = 0.57 mm/s, $|\Delta E_Q|_2$ = 0.71 mm/s (green).

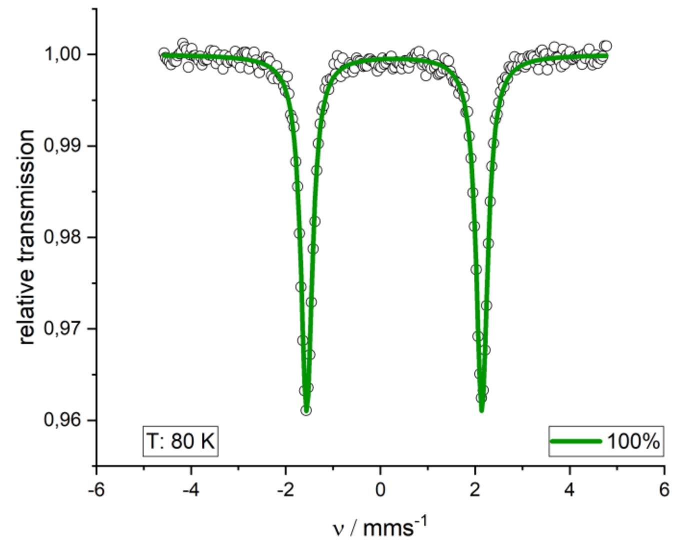

Figure S9. Zero field ^{57}Fe Mössbauer spectrum of solid $[2Fe–1S]^{4+}$ (**4**). The green line represents a fit with δ = 0.29 mm/s, $|\Delta E_Q|$ = 3.70 mm/s.

Magnetic Susceptibility Measurement

Temperature-dependent magnetic susceptibility measurements were carried out with a *Quantum-Design* MPMS3 SQUID magnetometer equipped with a 7 Tesla magnet in the range from 295/300 to 2.0 K at a magnetic field of 0.5 T. The powdered samples were contained in a polycarbonate capsule and fixed in a non-magnetic sample holder. Each raw data file for the measured magnetic moment was corrected for the diamagnetic contribution of the sample holder and the polycarbonate capsule. The molar susceptibility data were corrected for the diamagnetic contribution.

Simulation of the experimental magnetic data was performed with the *julX* program: E. Bill, Max-Planck Institute for Chemical Energy Conversion, Mülheim/Ruhr, Germany.

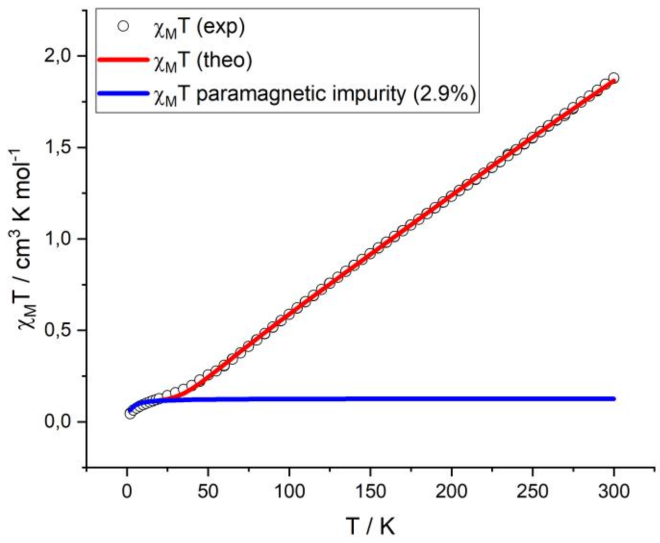

Figure S10. Plot of $\chi_M T$ vs. temperature for **2** with an applied field B = 0.5 T. The red line represents the best fit with the parameters $S_1 = S_2 = 2$, J = = -53 cm^{-1}, $g_1 = g_2 = 2.010$. The blue line represents a paramagnetic impurity PI (monomeric Iron (III), S = 5/2).

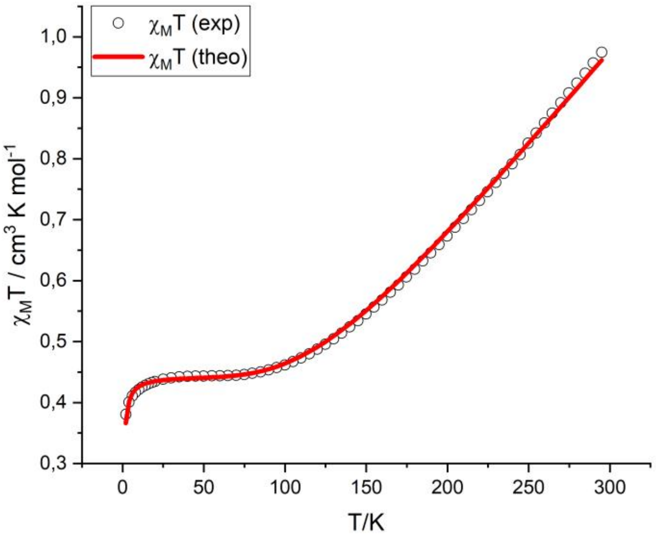

Figure S11. Plot of $\chi_M T$ vs. temperature for **3** with an applied field B = 0.5 T. The red line represents the best fit with the parameters $S_1 = 5/2$ and $S_2 = 2$, J = −116 cm^{-1}, $g_1 = 2.08$ $g_2 = 2.02$.

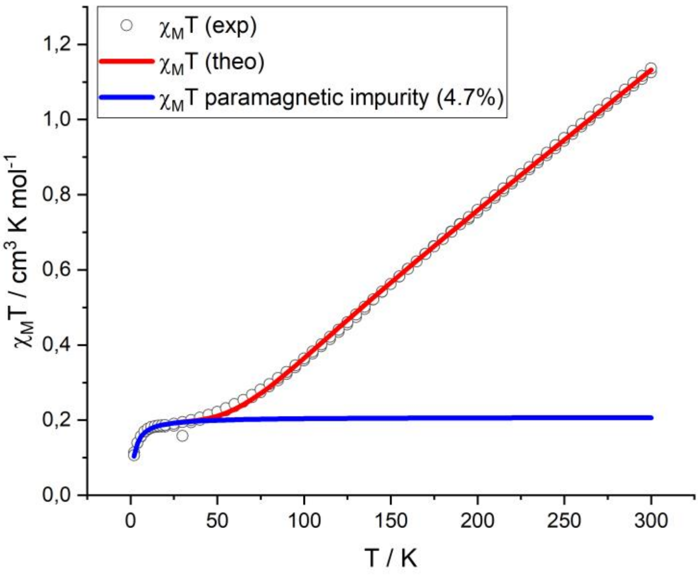

Figure S12. Plot of $\chi_M T$ vs. temperature for **4** with an applied field B = 0.5 T. The red line represents the best fit with the parameters $S_1 = S_2 = 5/2$, J = J = −98 cm^{-1}, $g_1 = g_2 = 2.083$. The blue line represents a paramagnetic impurity PI (monomeric Iron (III), S = 5/2).

X-Ray diffraction analysis and molecular structures

Data for **2** (CCDC 2048098), **3** (CCDC 2048164), **4** (CCDC 2048165), **5** (CCDC 2048166) and **6** (CCDC 2048167) were collected at 100 K on a Bruker Quest D8 diffractometer using a graphite-monochromated Mo-Kα radiation and equipped with an *Oxford Instrument Cooler Device*. The structures have been solved using either OLEX SHELXT V2014/1[5] and refined by means of least-squares procedures on a F^2 with the aid of the program SHELXL-2016/6. include in the softwares package WinGX version 1.63[6] or using CRYSTALS.

The Atomic Scattering Factors were taken from *International Tables for X-Ray Crystallography*[7]. All non-hydrogen atoms were refined anisotropically. All hydrogens atoms were refined by using a riding model. Absorption corrections were introduced by using the MULTISCAN and X-Red program. Drawings of molecules are performed with the program DIAMOND with 50% probability displacement ellipsoids for non-H atoms. Depiction of H atoms is omitted for clarity.

Table S3. Crystal data and structure refinement for **2**.

Empirical formula	$C_{100}H_{184}Fe_2K_2N_4O_{16}SSi_4$
Formula weight	2032.82
Temperature/K	100.0
Crystal system	triclinic
Space group	P-1
a/Å	16.9239(7)
b/Å	18.7019(7)
c/Å	19.8954(8)
α/°	89.747(2)
β/°	68.0070(10)
γ/°	77.8400(10)
Volume/Å^3	5688.5(4)
Z	2
ρ_{calc}g/cm^3	1.187
μ/mm^{-1}	0.447
F(000)	2204.0
Crystal size/mm^3	0.366 × 0.301 × 0.244
Radiation	MoKα (λ = 0.71073)
2Θ range for data collection/°	4.43 to 54.388
Index ranges	-21 ≤ h ≤ 21, -24 ≤ k ≤ 24, -25 ≤ l ≤ 25
Reflections collected	214747
Independent reflections	25259 [R_{int} = 0.0654, R_{sigma} = 0.0370]
Data/restraints/parameters	25259/0/1190
Goodness-of-fit on F^2	1.023
Final R indexes [I>=2σ (I)]	R_1 = 0.0370, wR_2 = 0.0759
Final R indexes [all data]	R_1 = 0.0574, wR_2 = 0.0828
Largest diff. peak/hole / e Å^{-3}	0.81/-0.49

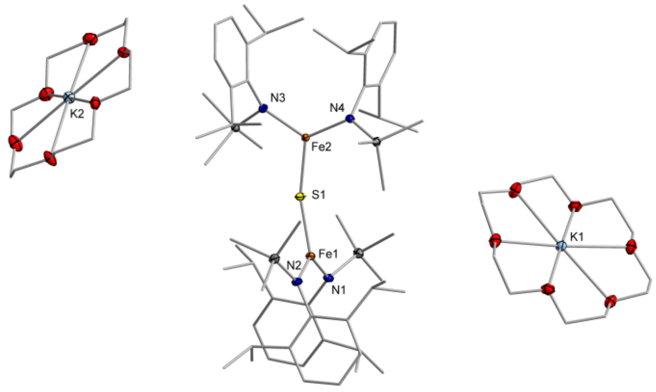

Figure S13. Molecular structure of **2** within the crystal. Hydrogen atoms cations have been omitted for clarity.

Table S4. Crystal data and structure refinement for **3**.

Empirical formula	$C_{73}H_{124.85}Fe_2KN_4O_{6.25}SSi_4$*
Formula weight	1453.83
Temperature/K	99.99
Crystal system	triclinic
Space group	P-1
a/Å	14.6545(7)
b/Å	19.1914(9)
c/Å	19.4547(9)
α/°	118.8410(10)
β/°	101.094(2)
γ/°	94.756(2)
Volume/Å^3	4603.9(4)
Z	2
ρ_{calc}g/cm^3	1.049
μ/mm^{-1}	0.478
F(000)	1568.0
Crystal size/mm^3	0.395 × 0.263 × 0.22
Radiation	MoKα (λ = 0.71073)
2Θ range for data collection/°	4.26 to 54.424
Index ranges	-18 ≤ h ≤ 18, -24 ≤ k ≤ 24, -24 ≤ l ≤ 25
Reflections collected	169519
Independent reflections	20466 [R_{int} = 0.0918, R_{sigma} = 0.0543]
Data/restraints/parameters	20466/138/948
Goodness-of-fit on F^2	1.082
Final R indexes [I>=2σ (I)]	R_1 = 0.0733, wR_2 = 0.1971
Final R indexes [all data]	R_1 = 0.1076, wR_2 = 0.2154
Largest diff. peak/hole / e Å^{-3}	1.58/-0.54

* The empirical formula differs from an integer number due to an Et_2O molecule with chemical occupancy of 25% in the asymmetrical unit.

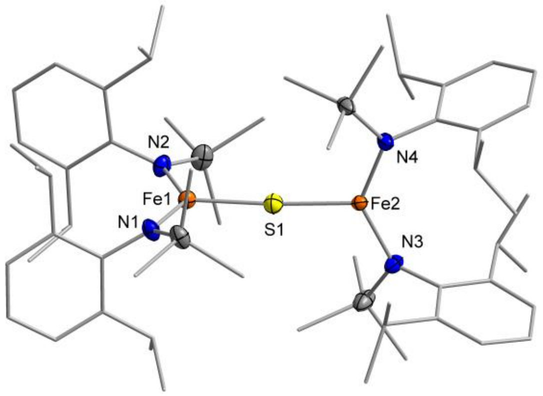

Figure S14. Molecular structure of **3** within the crystal. Hydrogen atoms and the [K$^+$(18-crown-6)] cation have been omitted for clarity.

Table S5. Crystal data and structure refinement for **4**.

Empirical formula	$C_{70}H_{128}Fe_2N_4SSi_4$
Formula weight	1281.88
Temperature/K	100.0
Crystal system	triclinic
Space group	P-1
a/Å	11.8424(6)
b/Å	14.9042(8)
c/Å	23.8758(12)
α/°	75.520(2)
β/°	85.210(2)
γ/°	68.222(2)
Volume/Å^3	3788.8(3)
Z	2
ρ_{calc}g/cm^3	1.124
μ/mm^{-1}	0.513
F(000)	1400.0
Crystal size/mm^3	0.251 × 0.228 × 0.117
Radiation	MoKα (λ = 0.71073)
2Θ range for data collection/°	4.5 to 54.344
Index ranges	-15 ≤ h ≤ 15, -19 ≤ k ≤ 19, -30 ≤ l ≤ 30
Reflections collected	135963
Independent reflections	16811 [R_{int} = 0.0558, R_{sigma} = 0.0334]
Data/restraints/parameters	16811/0/738
Goodness-of-fit on F^2	1.030
Final R indexes [I>=2σ (I)]	R_1 = 0.0500, wR_2 = 0.1174
Final R indexes [all data]	R_1 = 0.0685, wR_2 = 0.1270
Largest diff. peak/hole / e Å^{-3}	1.98/-1.06

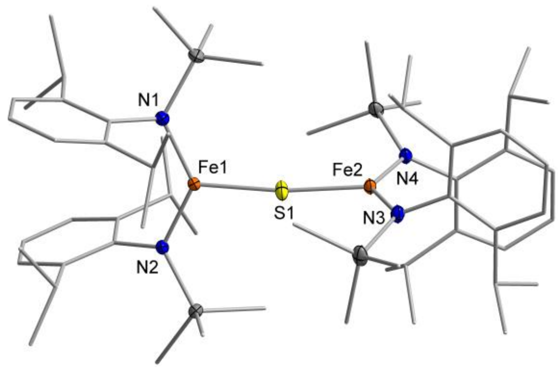

Figure S15. Molecular structure of **4** within the crystal. Hydrogen atoms are omitted for clarity.

Table S6. Crystal data and structure refinement for **5**.

Empirical formula	$C_{59}H_{108}FeK_2N_2O_{13}S_3Si_2$
Formula weight	1339.88
Temperature/K	100
Crystal system	monoclinic
Space group	$P2_1/n$
a/Å	13.0750(6)
b/Å	25.3112(11)
c/Å	25.1634(10)
α/°	90
β/°	102.486(2)
γ/°	90
Volume/Å^3	8130.7(6)
Z	4
ρ_{calc}g/cm^3	1.095
μ/mm^{-1}	0.443
F(000)	2880.0
Crystal size/mm^3	0.503 × 0.424 × 0.402
Radiation	MoKα (λ = 0.71073)
2Θ range for data collection/°	4.532 to 53.768
Index ranges	-16 ≤ h ≤ 16, -32 ≤ k ≤ 32, -31 ≤ l ≤ 31
Reflections collected	141659
Independent reflections	17502 [R_{int} = 0.0479, R_{sigma} = 0.0277]
Data/restraints/parameters	17502/0/753
Goodness-of-fit on F^2	1.023
Final R indexes [I>=2σ (I)]	R_1 = 0.0399, wR_2 = 0.0910
Final R indexes [all data]	R_1 = 0.0524, wR_2 = 0.0964
Largest diff. peak/hole / e Å^{-3}	0.47/-0.37

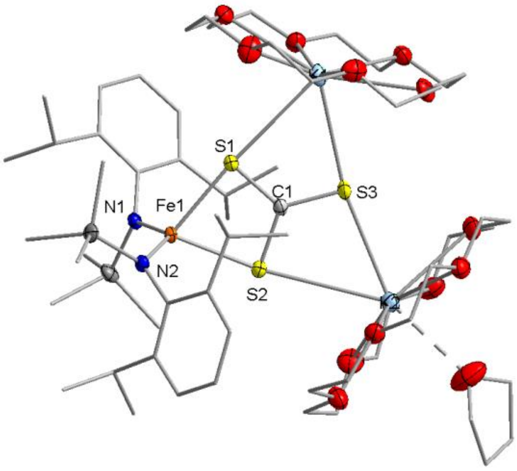

Figure S16. Molecular structure of **5** within the crystal. Hydrogen atoms have been omitted for clarity.

Table S7. Crystal data and structure refinement for **6**.

Empirical formula	$C_{47}H_{83}FeKN_2O_7S_3Si_2$
Formula weight	1035.46
Temperature/K	100.0
Crystal system	orthorhombic
Space group	$P2_12_12_1$
a/Å	9.9156(4)
b/Å	23.3884(10)
c/Å	24.4430(10)
α/°	90
β/°	90
γ/°	90
Volume/Å^3	5668.6(4)
Z	4
ρ_{calc}g/cm^3	1.213
μ/mm^{-1}	0.537
F(000)	2224.0
Crystal size/mm^3	0.488 × 0.22 × 0.115
Radiation	MoKα (λ = 0.71073)
2Θ range for data collection/°	4.434 to 49.996
Index ranges	-11 ≤ h ≤ 10, -27 ≤ k ≤ 27, -29 ≤ l ≤ 29
Reflections collected	58745
Independent reflections	9982 [R_{int} = 0.0341, R_{sigma} = 0.0223]
Data/restraints/parameters	9982/138/790
Goodness-of-fit on F^2	1.069
Final R indexes [I>=2σ (I)]	R_1 = 0.0291, wR_2 = 0.0683
Final R indexes [all data]	R_1 = 0.0313, wR_2 = 0.0692
Largest diff. peak/hole / e Å^{-3}	0.28/-0.26
Flack parameter	0.461(4)

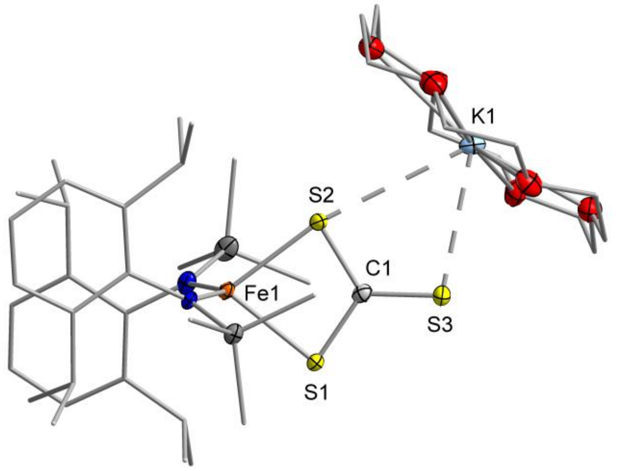

Figure S17. Molecular structure of **6** within the crystal. Hydrogen atoms have been omitted for clarity.

References

[1] E. M. Schubert, *J. Chem. Educ.* **1992**, *69*, 62.

[2] D. K. Kennepohl, S. Brooker, G. M. Sheldrick, H. W. Roesky, *Chem. Ber.* **1991**, *124*, 2223–2225.

[3] C. Y. Lin, J. D. Guo, J. C. Fettinger, S. Nagase, F. Grandjean, G. J. Long, N. F. Chilton, P. P. Power, *Inorg. Chem.* **2013**, *52*, 13584–13593.

[4] C. Y. Lin, J. C. Fettinger, F. Grandjean, G. J. Long, P. P. Power, *Inorg. Chem.* **2014**, *53*, 9400–9406.

[5] G. M. Sheldrick, *Acta Crystallogr. Sect. A Found. Crystallogr.* **2015**, *71*, 3–8.

[6] L. J. Farrugia, *J. Appl. Cryst.* **1999**, *32*, 837–838.

[7] Hamilton W. C., *International Tables for X-Ray Crystallography*, Kynoch Press, Birmingham, **1974**.
